# Supplementary material for: COVID-19 Vaccine Refusal and Delay among Adults in Italy: Evidence from the OBVIOUS Project, a National Survey in Italy
Source: Vaccines (Basel). 2023 Apr 13;11(4):839. doi: 10.3390/vaccines11040839 (PMC10141216; doi:10.3390/vaccines11040839)
Supplement: Supplementary file 1 [file vaccines-11-00839-s001.zip › vaccines-2298245-supplementary.pdf]

SUPPLEMENTARY MATERIAL

Table S1. Resident adult population of Italy by NUTS statistical region, gender, and age group at January 1, 2022 (*n* = 49 783 836).

Age group	Northwestern Italy		Northeastern Italy		Central Italy		Southern Italy		Insular Italy	
	Male	Female	Male	Female	Male	Female	Male	Female	Male	Female
18–24 y	553 033	505 245	409 914	374 523	402 087	368 995	532 647	494 803	239 009	220 219
25–34 y	836 151	791 227	613 449	576 453	611 746	580 657	779 592	755 610	355 025	344 717
35–44 y	969 032	951 652	703 399	695 075	727 507	735 948	844 648	850 252	395 793	398 899
45–54 y	1 283 458	1 277 029	941 466	938 983	930 711	976 955	1 001 115	1 046 563	482 219	499 758
55–64 y	1 157 096	1 199 031	852 267	879 622	844 991	908 707	941 859	1 015 616	453 686	489 038
≥65 y	1 687 998	2 201 703	1 221 291	1 561 980	1 250 238	1 623 659	1 331 520	1 673 237	658 804	835 929

Source: Italian National Institute of Statistics (Istat).

Notes: Northwestern Italy includes the regions of Piedmont, Aosta Valley, Lombardy, and Liguria; Northeastern Italy includes the regions of Trentino-South Tyrol, Veneto, Friuli-Venezia Giulia, and Emilia-Romagna; Central Italy includes the regions of Tuscany, Umbria, Marche, and Lazio; Southern Italy includes the regions of Abruzzo, Molise, Campania, Apulia, Basilicata, and Calabria; Insular Italy includes the regions of Sicily and Sardinia.

Table S2. Relative distribution of the OBVIOUS sample (n = 10 000) and Italy's adult population at January 1, 2022 (n = 49 783 836) by NUTS statistical region, gender, and age group. Discrepancies between observed weights and expected weights to be obtained via stratified sampling are quantified as the difference between the relative contributions of each stratum in the study sample vs. the target population.

	Northwestern Italy		Northeastern Italy		Central Italy		Southern Italy		Insular Italy	
	Male	Female*	Male	Female*	Male	Female*	Male	Female*	Male	Female*
Study sample (*Observed*)										
18–24 y	0.00850	0.01140	0.01000	0.01030	0.00910	0.01020	0.01440	0.01510	0.00440	0.00470
25–34 y	0.01530	0.02040	0.01570	0.01950	0.01500	0.01920	0.02560	0.02040	0.00670	0.01190
35–44 y	0.02830	0.03400	0.01760	0.02100	0.01980	0.02170	0.02510	0.02500	0.01070	0.01620
45–54 y	0.02990	0.03120	0.01590	0.01990	0.01960	0.02100	0.01900	0.02340	0.00950	0.01440
55–64 y	0.02470	0.02650	0.01240	0.01600	0.01870	0.02000	0.01390	0.01650	0.00750	0.00950
≥65 y	0.02400	0.02080	0.01790	0.00990	0.01540	0.01060	0.01810	0.01300	0.00690	0.00670
Italian population (*Expected*)										
18–24 y	0.01111	0.01015	0.00823	0.00752	0.00808	0.00741	0.01070	0.00994	0.00480	0.00442
25–34 y	0.01680	0.01589	0.01232	0.01158	0.01229	0.01166	0.01566	0.01518	0.00713	0.00692
35–44 y	0.01946	0.01912	0.01413	0.01396	0.01461	0.01478	0.01697	0.01708	0.00795	0.00801
45–54 y	0.02578	0.02565	0.01891	0.01886	0.01870	0.01962	0.02011	0.02102	0.00969	0.01004
55–64 y	0.02324	0.02408	0.01712	0.01767	0.01697	0.01825	0.01892	0.02040	0.00911	0.00982
≥65 y	0.03391	0.04423	0.02453	0.03138	0.02511	0.03261	0.02675	0.03361	0.01323	0.01679
O−E difference										
18–24 y	−0.00261	0.00125	0.00177	0.00278	0.00102	0.00279	0.00370	0.00516	−0.00040	0.00028
25–34 y	−0.00150	0.00451	0.00338	0.00792	0.00271	0.00754	0.00994	0.00522	−0.00043	0.00498
35–44 y	0.00884	0.01488	0.00347	0.00704	0.00519	0.00692	0.00813	0.00792	0.00275	0.00819
45–54 y	0.00412	0.00555	−0.00301	0.00104	0.00090	0.00138	−0.00111	0.00238	−0.00019	0.00436
55–64 y	0.00146	0.00242	−0.00472	−0.00167	0.00173	0.00175	−0.00502	−0.00390	−0.00161	−0.00032
≥65 y	−0.00991	−0.02343	−0.00663	−0.02148	−0.00971	−0.02201	−0.00865	−0.02061	−0.00633	−0.01009

*Including non-binary individuals and respondents who preferred not to disclose their gender (24 out of 10 000).

Notes: Northwestern Italy includes the regions of Piedmont, Aosta Valley, Lombardy, and Liguria; Northeastern Italy includes the regions of Trentino-South Tyrol, Veneto, Friuli-Venezia Giulia, and Emilia-Romagna; Central Italy includes the regions of Tuscany, Umbria, Marche, and Lazio; Southern Italy includes the regions of Abruzzo, Molise, Campania, Apulia, Basilicata, and Calabria; Insular Italy includes the regions of Sicily and Sardinia.

OBVIOUS, Observatory on Vaccine Hesitancy in Italy - Online UNIBO Surveys.

Table S3. Sociodemographic characteristics of the study sample, overall and by COVID-19 vaccine uptake status. Counts and percentages are unweighted, that is, not adjusted via post-stratification.

Characteristic	All (n = 10 000)	Got It as Soon as Possible (n = 8202)	Put Off Getting It (n = 897)	Refused to Get It (n = 687)	Vaccine-Exempt (n = 214)	P-Value
Gender						<0.001
Male	4796 (100.0%)	3981 (83.0%)	391 (8.2%)	285 (5.9%)(−)	139 (2.9%)(+)	
Female	5180 (100.0%)	4204 (81.2%)	504 (9.7%)	398 (7.7%)(+)	74 (1.4%)(−)	
Non-binary	17 (100.0%)	12 (70.6%)	1 (5.9%)	4 (23.5%)	0 (0.0%)	
Prefer not to say	7 (100.0%)	5 (71.4%)	1 (14.3%)	0 (0.0%)	1 (14.3%)	
Age group, y						<0.001
18–24	981 (100.0%)	848 (86.4%)(+)	72 (7.3%)	35 (3.6%)(−)	26 (2.7%)	
25–34	1697 (100.0%)	1349 (79.5%)	209 (12.3%)(+)	96 (5.7%)	43 (2.5%)	
35–44	2194 (100.0%)	1692 (77.1%)(−)	245 (11.2%)(+)	194 (8.8%)(+)	63 (2.9%)	
45–54	2038 (100.0%)	1652 (81.1%)	180 (8.8%)	173 (8.5%)	33 (1.6%)	
55–64	1657 (100.0%)	1410 (85.1%)(+)	132 (8.0%)	97 (5.9%)	18 (1.1%)	
≥65	1433 (100.0%)	1251 (87.3%)(+)	59 (4.1%)(−)	92 (6.4%)	31 (2.2%)	
Place of Residence Degree of Urbanization						<0.001
City (densely populated area)	3881 (100.0%)	3247 (83.7%)(+)	319 (8.2%)	249 (6.4%)	66 (1.7%)	
Town or suburb (intermediate density area)	4685 (100.0%)	3868 (82.6%)	410 (8.8%)	338 (7.2%)	69 (1.5%)(−)	
Rural area (thinly populated area)	1434 (100.0%)	1087 (75.8%)(−)	168 (11.7%)(+)	100 (7.0%)	79 (5.5%)(+)	
Educational attainment						<0.001
Less than high school diploma	1341 (100.0%)	1080 (80.5%)	119 (8.9%)	131 (9.8%)(+)	11 (0.8%)(−)	
High school diploma	5714 (100.0%)	4692 (82.1%)	521 (9.1%)	412 (7.2%)	89 (1.6%)(−)	
Academic degree	2119 (100.0%)	1817 (85.7%)(+)	155 (7.3%)	107 (5.0%)(−)	40 (1.9%)	
Post-graduate/Doctorate degree	826 (100.0%)	613 (74.2%)(−)	102 (12.3%)(+)	37 (4.5%)	74 (9.0%)(+)	
Opinion on vaccination policies						<0.001
Governments should restrict themselves to making vaccines available and providing information, without influencing individual decisions	3720 (100.0%)	2495 (67.1%)(−)	547 (14.7%)(+)	556 (14.9%)(+)	122 (3.3%)(+)	
Governments must make vaccines mandatory during health emergencies	3432 (100.0%)	3320 (96.7%)(+)	82 (2.4%)(−)	19 (0.6%)(−)	11 (0.3%)(−)	
Governments must offer incentives to those who get vaccinated, but without imposing any vaccine mandate	2089 (100.0%)	1692 (81.0%)	232 (11.1%)(+)	98 (4.7%)(−)	67 (3.2%)(+)	
Governments must use penalties for those who decide not to get vaccinated	759 (100.0%)	695 (91.6%)(+)	36 (4.7%)(−)	14 (1.8%)(−)	14 (1.8%)	
Use of homeopathy, naturopathy, chiropractic or osteopathy as the main source of care						<0.001

Yes	1502 (100.0%)	956 (63.6%)(−)	245 (16.3%)(+)	193 (12.8%)(+)	108 (7.2%)(+)	
No	8498 (100.0%)	7246 (85.3%)(+)	652 (7.7%)(−)	494 (5.8%)(−)	106 (1.2%)(−)	
Visit to one of the following practitioners over the last year						<0.001
Osteopath	808 (100.0%)	655 (81.1%)	91 (11.3%)	37 (4.6%)	25 (3.1%)	
Homeopath	414 (100.0%)	259 (62.6%)(−)	74 (17.9%)(+)	43 (10.4%)	38 (9.2%)(+)	
Naturopath	255 (100.0%)	153 (60.0%)(−)	46 (18.0%)(+)	28 (11.0%)	28 (11.0%)(+)	
Chiropractor	232 (100.0%)	158 (68.1%)(−)	34 (14.7%)	15 (6.5%)	25 (10.8%)(+)	
None of the above	8291 (100.0%)	6977 (84.2%)(+)	652 (7.9%)(−)	564 (6.8%)	98 (1.2%)(−)	
Use of one of the following products over the last year						<0.001
Supplements (e.g., proteins, vitamins, minerals)	5298 (100.0%)	4391 (82.9%)	451 (8.5%)	378 (7.1%)	78 (1.5%)(−)	
Herbal remedies	799 (100.0%)	599 (75.0%)(−)	105 (13.1%)(+)	64 (8.0%)	31 (3.9%)(+)	
Homeopathic products	450 (100.0%)	296 (65.8%)(−)	78 (17.3%)(+)	38 (8.4%)	38 (8.4%)(+)	
Aromatherapy (e.g., essential oils)	270 (100.0%)	174 (64.4%)(−)	42 (15.6%)(+)	23 (8.5%)	31 (11.5%)(+)	
None of the above	3183 (100.0%)	2742 (86.1%)(+)	221 (6.9%)(−)	184 (5.8%)	36 (1.1%)(−)	

(+)Adjusted residual ≥3.29 (≥$z_{0.001/2}$), that is, frequency is significantly greater than what would be expected if the null hypothesis of independence was true.

(−)Adjusted residual ≤3.29 (≤$z_{0.001/2}$), that is, frequency is significantly lower than what would be expected if the null hypothesis of independence was true.

COVID-19, coronavirus disease 2019.

Table S4. Reasons for getting COVID-19 vaccination as soon as possible, putting off getting it, and refusing to get it. Counts and percentages are unweighted, that is, not adjusted via post-stratification.

Answer	All	Importance Ranking			
		1st	2nd	3rd	>3rd
Got it as soon as possible					
Avoiding severe illness	8202 (100.0%)	4320 (52.7%)	962 (11.7%)	717 (8.7%)	2203 (26.9%)
Protecting dear ones	8202 (100.0%)	1760 (21.5%)	2963 (36.1%)	1001 (12.2%)	2478 (30.2%)
Sense of civic duty	8202 (100.0%)	991 (12.1%)	1321 (16.1%)	1946 (23.7%)	3944 (48.1%)
Returning to normal life	8202 (100.0%)	814 (9.9%)	744 (9.1%)	1704 (20.8%)	4940 (60.2%)
Professional ethics	8202 (100.0%)	317 (3.9%)	343 (4.2%)	514 (6.3%)	7028 (85.7%)
Put off getting it					
Fear of side effects	897 (100.0%)	487 (54.3%)	107 (11.9%)	44 (4.9%)	259 (28.9%)
Getting more info about safety	897 (100.0%)	239 (26.6%)	267 (29.8%)	55 (6.1%)	336 (37.5%)
Lack of time and/or difficulties of access	897 (100.0%)	83 (9.3%)	42 (4.7%)	76 (8.5%)	696 (77.6%)
Opposition to vaccines in general	897 (100.0%)	45 (5.0%)	74 (8.2%)	76 (8.5%)	702 (78.3%)
COVID-19 is not a serious disease	897 (100.0%)	28 (3.1%)	53 (5.9%)	85 (9.5%)	731 (81.5%)
Protest against government	897 (100.0%)	15 (1.7%)	22 (2.5%)	48 (5.4%)	812 (90.5%)
Refused to get it					
Fear of side effects	687 (100.0%)	384 (55.9%)	106 (15.4%)	44 (6.4%)	153 (22.3%)
Lack of information about safety	687 (100.0%)	183 (26.6%)	256 (37.3%)	48 (7.0%)	200 (29.1%)
COVID-19 is not a serious disease	687 (100.0%)	46 (6.7%)	29 (4.2%)	99 (14.4%)	513 (74.7%)
Opposition to vaccines in general	687 (100.0%)	31 (4.5%)	29 (4.2%)	56 (8.2%)	571 (83.1%)
Fear of needles and/or doctors	687 (100.0%)	25 (3.6%)	17 (2.5%)	15 (2.2%)	630 (91.7%)
Protest against government	687 (100.0%)	18 (2.6%)	27 (3.9%)	64 (9.3%)	578 (84.1%)

Notes: Respondents were asked to pick three reasons in order of importance.
COVID-19, coronavirus disease 2019.

Figure S1. COVID-19 vaccine uptake in the study sample (n = 10 000) between January 2021 and April/May 2022, overall and by NUTS statistical region. Percentages are unweighted, that is, not adjusted via post-stratification.

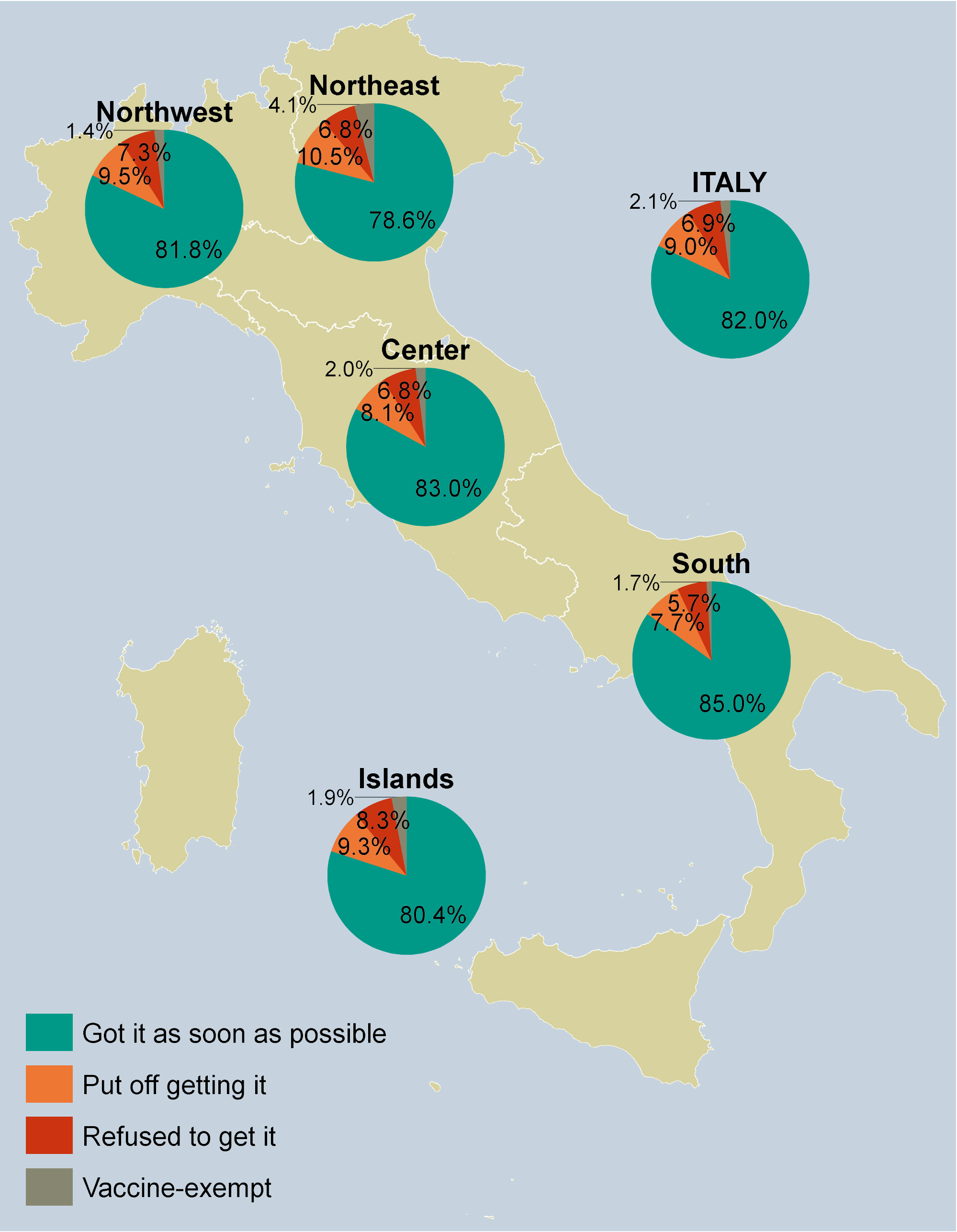

Notes: Northwestern Italy includes Piedmont, Aosta Valley, Lombardy, and Liguria; Northeastern Italy includes Trentino-South Tyrol, Veneto, Friuli-Venezia Giulia, and Emilia-Romagna; Central Italy includes Tuscany, Umbria, Marche, and Lazio; Southern Italy includes Abruzzo, Molise, Campania, Apulia, Basilicata, and Calabria; Insular Italy includes Sicily and Sardinia.

COVID-19, coronavirus disease 2019.

Figure S2. Trust in science scored on a scale from a minimum of 1 to a maximum of 10, by COVID-19 vaccine uptake status. Percentages are unweighted, that is, not adjusted via post-stratification.

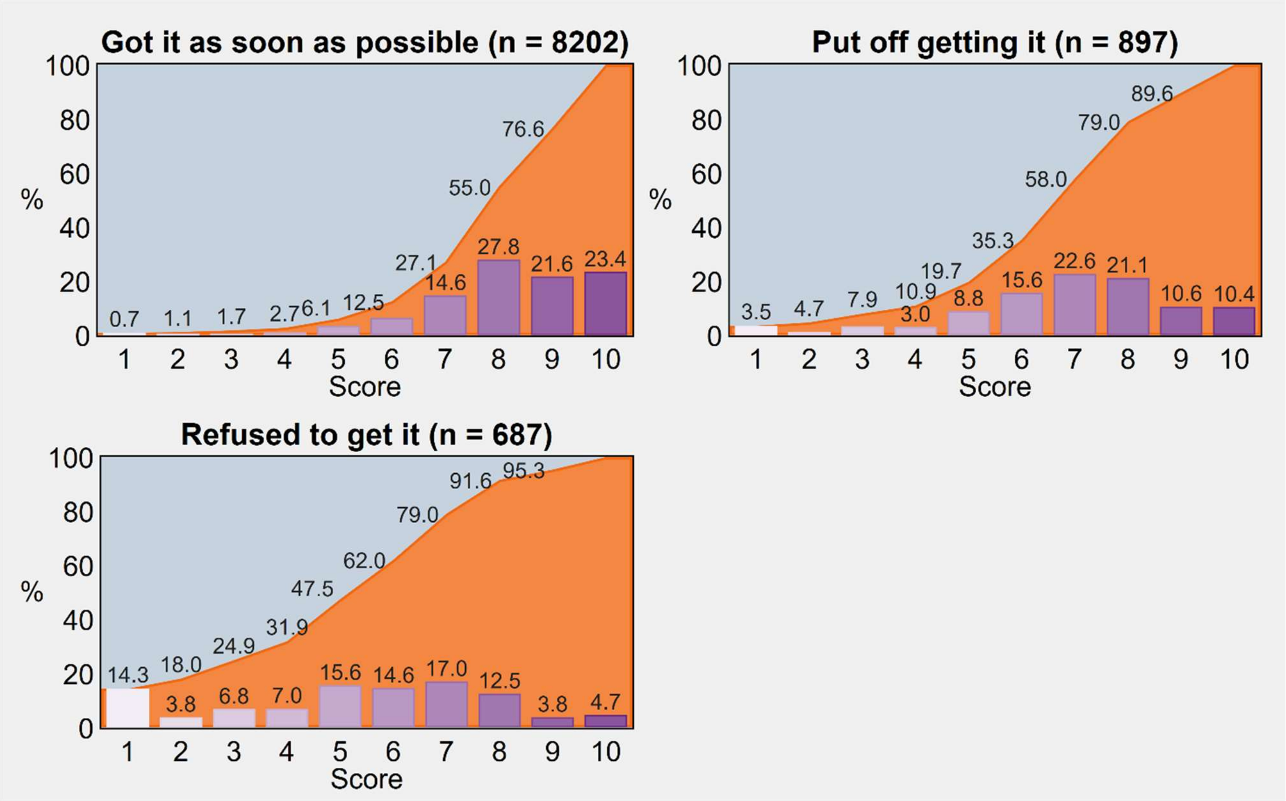

Notes: Cumulative frequencies are displayed with the aid of area charts in the background. Trust among vaccine-exempt individuals is not displayed due to small numbers (n = 214).
COVID-19, coronavirus disease 2019.

Figure S3. Trust in the Italian government scored on a scale from a minimum of 1 to a maximum of 10, by COVID-19 vaccine uptake status. Percentages are unweighted, that is, not adjusted via post-stratification.

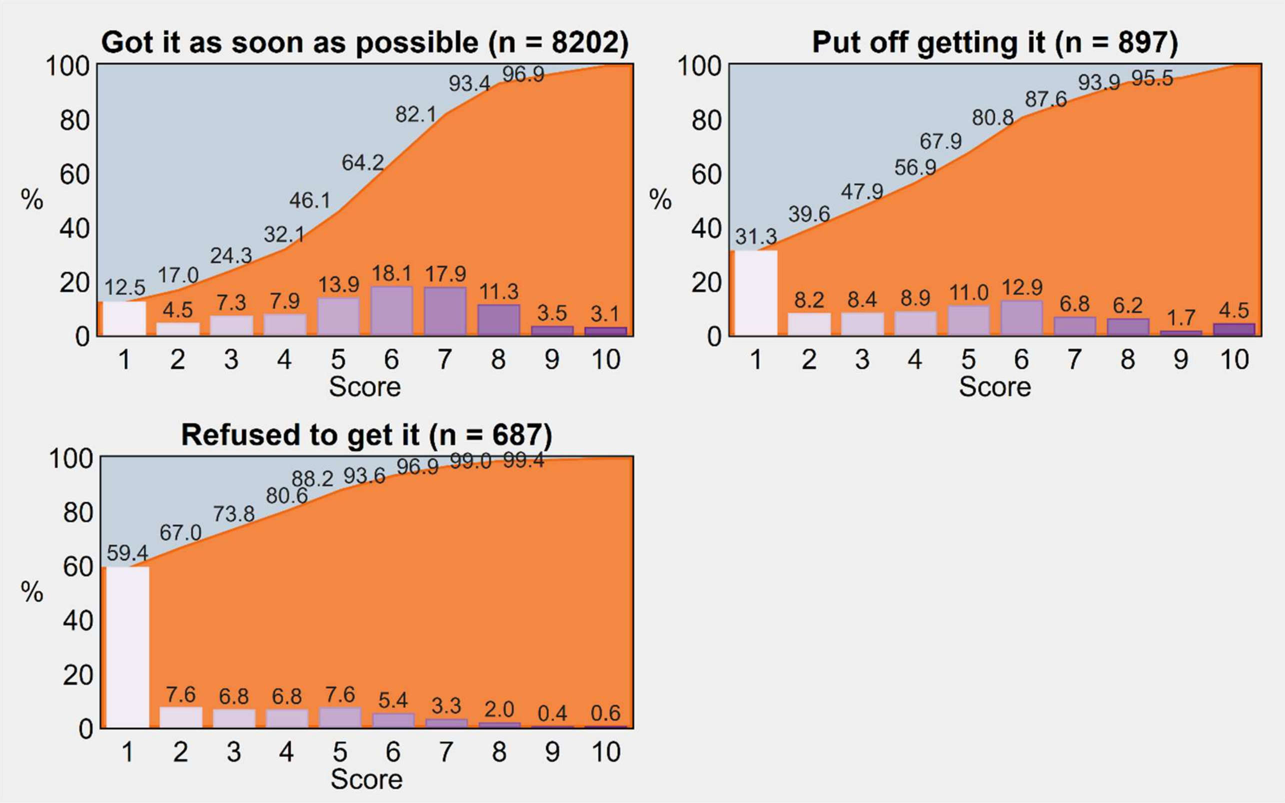

Notes: Cumulative frequencies are displayed with the aid of area charts in the background. Trust among vaccine-exempt individuals is not displayed due to small numbers (n = 214).
COVID-19, coronavirus disease 2019.

Figure S4. Voting intention as of April/May 2022 by COVID-19 vaccine uptake status. Percentages are unweighted, that is, not adjusted via post-stratification.

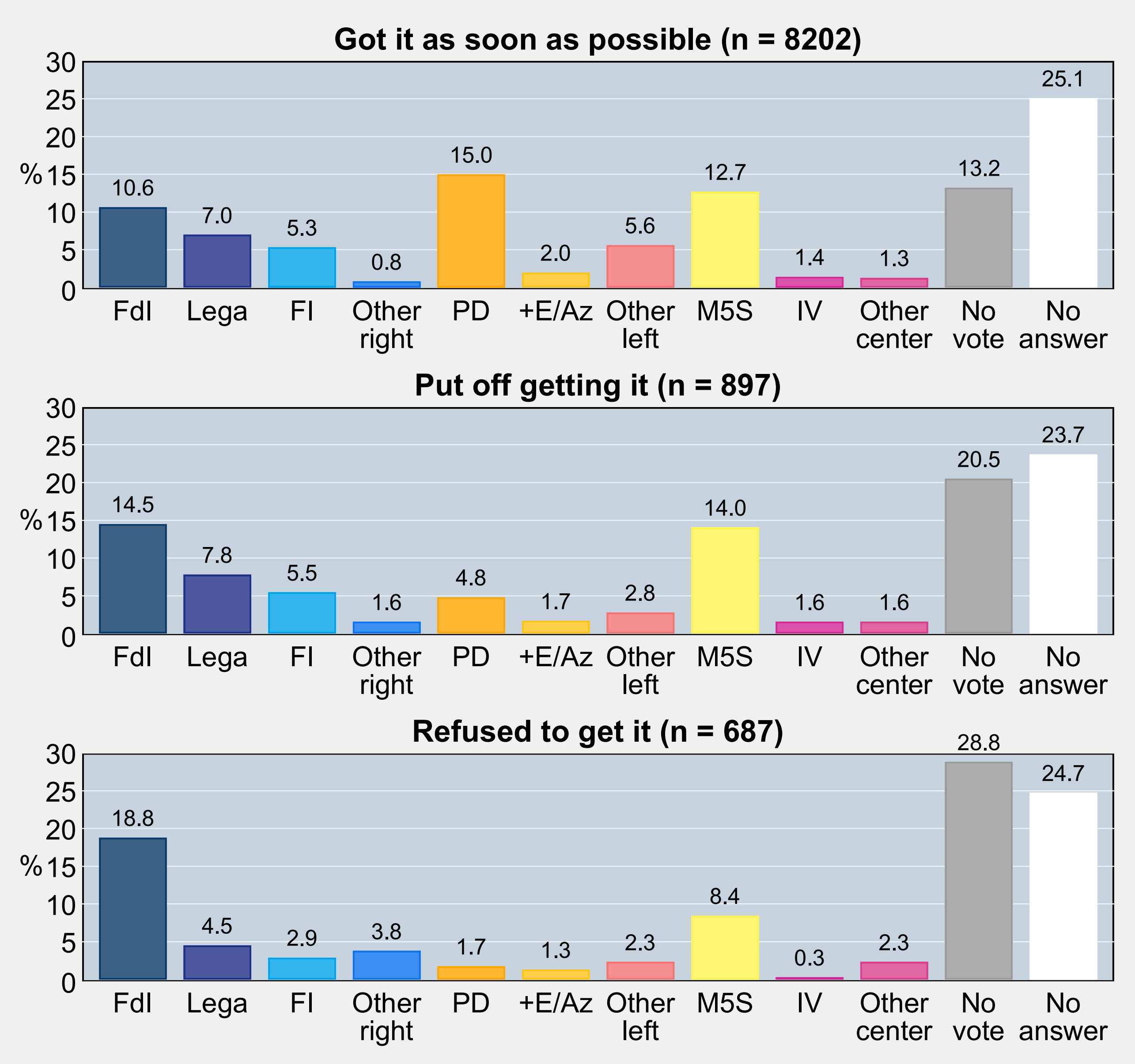

Notes: Voting intention among vaccine-exempt individuals is not displayed due to small numbers (n = 214).
FdI, Fratelli d'Italia (Brothers of Italy); *FI, Forza Italia* (Forward Italy); *PD, Partito Democratico* (Democratic Party); *+E, Più Europa* (More Europe); *Az, Azione* (Action); *M5S, Movimento 5 Stelle* (Five Star Movement); *IV, Italia Viva* (Italy Alive).
